# Metabolic profile in elite badminton match play and training drills

**DOI:** 10.1002/ejsc.12196

**Published:** 2024-10-12

**Authors:** Antonia Edel, Jo‐Lâm Vuong, Sebastian Kaufmann, Olaf Hoos, Thimo Wiewelhove, Alexander Ferrauti

**Affiliations:** ^1^ Ruhr University Bochum Bochum Germany; ^2^ Julius‐Maximilians‐Universität Würzburg Würzburg Germany; ^3^ IST University of Applied Sciences Düsseldorf Germany

**Keywords:** metabolism, modeling, performance, physiology, training

## Abstract

Aim of the study was to analyze the metabolic profile of badminton matches and training drills. Therefore, 11 male (23.2 ± 3.8 years, 182 ± 7 cm, 74.4 ± 8.4 kg) and five female (19.3 ± 1.5 years, 170 ± 6 cm, 62.6 ± 9.2 kg) elite badminton players participated in either a training match (*T*_*M*_; *n* = 7) and/or three protocols of multifeeding drills (*T*_10_, *T*_30,_ *T*_50;_ *n* = 13), that varied in interval and rest durations (10 s/10 s, 30 s/30 s, 50 s/50 s). Absolute and relative energetic costs (*W*_tot_ and *E*_tot_) and contribution to oxidative (*W*_Oxid_), phosphagen (*W*_PCr_), and anaerobic glycolytic (*W*_La_) metabolism were calculated by the three‐component PCr‐La‐O_2_‐method based on an indirect calorimetric approach from oxygen consumption during exercise, post exercise, and net blood lactate concentration. A novel intermittent approach was used to consider replenishment of phosphocreatine during each resting phase. Results show that during *T*_*M*_, *E*_tot_ was 676 ± 98J·kg^−1^ min^−1^, while metabolic pathways contributed by 56.9 ± 8.6% (*W*_Oxid_), 42.7 ± 8.7% (*W*_PCr_), and 0.4 ± 0.6% (*W*_La_). In the multifeeding drills *E*_tot_ was comparable between *T*_10_ (1020 ± 160J·kg^−1^ min^−1^) and *T*_30_ (985 ± 173 J·kg^−1^ min^−1^) but higher in *T*_50_ (1266 ± 194J·kg^−1^ min^−1^) (*p* < 0.001). Relative contribution of *W*_Oxid_ was lower in *T*_10_ (47.3 ± 7.7%) but similar in *T*_30_ (56.5 ± 6.2%) and *T*_50_ (57.3 ± 6.0%) (*p* < 0.001). *W*_PCr_ was highest in *T*_10_ (51.1 ± 8.3%) followed by *T*_30_ (42.2 ± 6.9%) and lowest in *T*_50_ (31.2 ± 7.7%) (*p* < 0.001). *W*_La_ was similar between *T*_10_ (1.6 ± 1.0%) and *T*_30_ (2.1 ± 1.0%) but higher in *T*_50_ (11.6 ± 4.8%) (*p* < 0.001). Concludingly, metabolic costs in badminton are predominantly covered by oxidative and phosphagen energetic pathways. Metabolic profiles of the multifeeding drills differ depending on rally/interval duration, with increasing contribution of anaerobic glycolysis and decreasing phosphagen contribution in case of longer intervals.

## Highlights

- This is the first study using an intermittent approach of the three‐component PCr‐La‐O_2_‐method, to determine the metabolic profile of elite badminton match play and the impact of drill prescription on metabolic stress in badminton‐specific training drills.
- The present findings reveal that energy in badminton generally is predominantly derived from oxidative and phosphagen energetic pathways, with the anaerobic glycolytic system contributing only minimally.
- Multifeeding drills essentially mirror the metabolic demands of matches but exhibit variations depending on rally and rest periods. These findings underscore the need to select training protocols thoughtfully, tailor training according to individualized goals and to structure those goals within a long‐term training periodization.

## 1 INTRODUCTION

Badminton stands out as a high‐paced and exceptionally demanding sport, necessitating elevated levels of physiological and physical fitness to compete successfully at the elite level. This is characterized by brief yet intense bursts of activity, including rapid accelerations and decelerations, frequent changes of direction, multiple jumps, and a variety of strokes that alternate with recovery phases of variable durations (Edel et al., 2019; Faude et al., 2007; Phomsoupha et al., 2015). In contrast to more predictable continuous types of exercise, badminton's acyclic and intermittent structure involves a complex and variable interplay between aerobic (mitochondrial) and anaerobic (non‐mitochondrial) energy systems (Dunst, 2020; Latzel et al., 2018). Understanding these intricate interactions during match play is crucial in identifying performance‐relevant requirements and aligning training contents accordingly (Zagatto et al., 2010). Moreover, gaining insight into metabolic stress induced by on‐court training drills is essential in designing well‐considered and targeted training programs. This involves understanding how adjustments in drill prescriptions, such as interval or rest durations, impact the energetic demands addressed (Ferrauti et al., 2001). Despite the significance of this knowledge, there is limited evidence regarding energetic demands in badminton. Thus, conditioning training in badminton is still based on educated guesses rather than on evidence‐based knowledge about the metabolic processes involved. Closing this gap is essential to refine training approaches and optimize performance in this dynamic and challenging sport.

In examining the temporal and notational profiles of badminton matches, earlier studies have shown that the average rally duration ranges from 6 to 12 s and the rally‐to‐rest ratio averages around 1:2. Based on physiological responses, such as oxygen consumption ($\dot{V}$ O_2_), heart rate (HR), and blood lactate (La) concentrations, previous research has suggested a metabolic profile of approximately 70% aerobic and 30% anaerobic energy supply, with the lactic component playing a subordinate role (Faude et al., 2007; Phomsoupha et al., 2015). However, recent studies have broken new ground by employing a three‐component model (PCr‐La‐O_2_) to estimate the actual amounts of aerobic (oxidative), anaerobic alactic (phosphagen), and lactic (anaerobic glycolytic) energy supply in different racquet sports (Fu et al., 2021; Milioni et al., 2018; Zagatto et al., 2016). This method relies on an indirect calorimetric approach, incorporating measurements of exercising $\dot{V}$ O_2_, post‐exercise $\dot{V}$ O_2_ (EPOC), and net La (Beneke et al., 2004; Milioni et al., 2018; Zagatto et al., 2016). In contrast to prior assumptions, one of these studies has reported a substantially different metabolic profile for badminton match play, revealing the dominance of the oxidative energy supply at 94.2%, with 4.4% phosphagen and 1.4% anaerobic glycolytic energy supply (Fu et al., 2021).

Given the intense nature of rallies in badminton and drawing parallels with findings in sports exhibiting similar activity profiles (Dunst, 2020), the reported low contribution of anaerobic energy systems raises questions. However, the recent study adopted a continuous calculation model, which may have contributed to these surprising findings by neglecting factors such as the replenishment of phosphocreatine (PCr) during resting phases and continuous La breakdown. Aligning with earlier investigations in sports such as basketball and squash, that employed both continuous and intermittent models, it seems reasonable that the continuous PCr‐La‐O_2_‐model significantly underestimates the share of anaerobic energy supply in intermittent exercises (Dunst, 2020; Latzel et al., 2018). Consequently, the metabolic profile of badminton remains unclear. In addition, there is hardly any evidence regarding the energetics of typical badminton‐specific drills, especially not for systematic variation in drill prescription. Using an intermittent approach of the three‐component PCr‐La‐O_2_‐method, the main objective of the present investigation was (1) to determine the metabolic profiles of elite badminton match play and specific on‐court conditioning drills and (2) to consider the impact of variations in the duration of intervals and rest periods on the contributions of oxidative, phosphagen, and glycolytic energy systems.

## 2 MATERIAL AND METHODS

### 2.1 Participants

Sixteen elite badminton players (eleven males: 23.2 ± 3.8 years, 182 ± 7 cm, 74.4 ± 8.4 kg; five females: 19.3 ± 1.5 years, 170 ± 6 cm, 62.6 ± 9.2 kg) participated in either a match (*n* = 3 [two males, one female]) or training (*n* = 9 [six males, three females]) analysis or in both (*n* = 4 [three males, one female]). All players were members of the German Olympic, perspective, or junior squad and all were part of the same training group. Before the study started, all players were informed about the testing procedures, the data policy, and the potential risks of participating in the study and gave their written informed consent to voluntarily participate. The players were instructed to maintain a regular diet and not perform any additional vigorous exercise prior to the evaluation. As the weekly training structures were comparable, the players were allowed to continue their usual training routines. The study design, procedures, and measurements aligned with the Declaration of Helsinki and were approved by the local ethics committee (EKSV21/2019).

### 2.2 Study design and procedures

**FIGURE 1:**
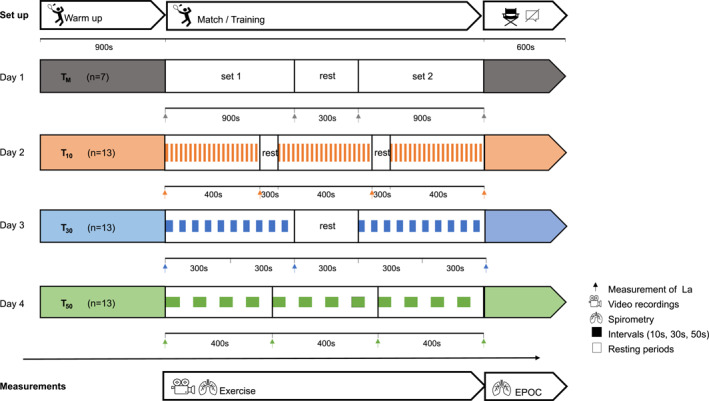
Overall study design, test set up, match (*T_M_*) and training protocols (*T*_10_, *T*_30_, *T*_50_) and measurements (*n*, sample size; La, blood lactate concentration; EPOC, post exercise oxygen consumption).

Based on a cross‐sectional study design, players engaged either in a training match (*T*_*M*_) and/or in three drills (*T*_10_, *T*_30_, and *T*_50_) of badminton multifeeding, on separate training days. *T*_*M*_ consisted of two 15 min sets with a 5 min break, arranged according to the official rules of the Badminton World Federation. Opponents were matched according to sex and performance level. Due to the temporal prescription, matches were continued if a score of 21 was exceeded. In the multifeeding drills the coach fed shuttlecocks with no breaks and in random order from the center of one side of the net to the player on the other side (Edel et al., 2023). The players aimed to perform high‐quality returns of each ball. This exercise is typical for on‐court conditioning in badminton. The active training time and rally‐to‐rest ratios in the drills were identical, but the protocols varied in terms of the duration of intervals and rest periods. Detailed protocol descriptions are provided in Table 1. Portable spirometry was used to monitor respiratory responses, blood samples were taken at predefined times (see Figure 1) to measure La, and video analysis was used to estimate the number and durations of the rally and resting phases as well as to count the number of strokes. For estimation of EPOC, players were advised to sit down with their arms resting on their lap while avoiding any movement and not speaking for 10 min immediately following the last rally. All tests took place on the same training court, on the same day of the week, and at similar times. Prior to each test, all players performed a 15 min warm‐up routine and spirometry was calibrated with a 3 L gas flow pump and a standard gas (oxygen [O_2_], 15.00%; carbon dioxide [CO_2_], 5.09%). The study design is illustrated in Figure 1.

**TABLE 1:** Protocol prescription of the multifeeding training drills (*T*_10_, *T*_30_, and *T*_50_).

	Number of series	Number of intervals	Interval duration [s]	Resting duration [s]	Rest between series [min]	Interval to rest ratio	Active playing time [min]	Total playing time [min]
*T*_10_	3	20	10	10	5	1:1	10	30
*T*_30_	2	10	30	30	5	1:1	10	25
*T*_50_	1	12	50	50	–	1:1	10	20

### 2.3 Measurements

Inspired and expired air was analyzed for O_2_ and CO_2_ concentrations using electrochemical cells and nondispersive infrared spectrometry. Ventilation was digitally recorded using Triple‐V sensor technology. Therefore, players carried a mobile spirometry system (MetaMax 3B–R2; Cortex Biophysik GmbH) on their chests. Breath‐by‐breath raw data were processed using a moving average of 1 s. La was determined from capillary blood using enzymatic amperometry. Blood samples were taken prior to and directly after each set from the right earlobe using 20 μL capillary tubes, hemolyzed in 1 mL micro test tubes, and analyzed for La using a Biosen S‐Line (EKF‐Diagnostik GmbH). All sessions were recorded with a video camera (LEGRIA HF G3; Canon Deutschland GmbH), HR was measured using chest belts (H10 Sensor; Polar Electro GmbH Deutschland), and the players were asked to rate their perceived exertion (RPE) after each set. Sufficient validity and reliability for field‐based testing has been previously reported for the spirometry device (Vogler et al., 2010).

### 2.4 Calculations

**FIGURE 2:**
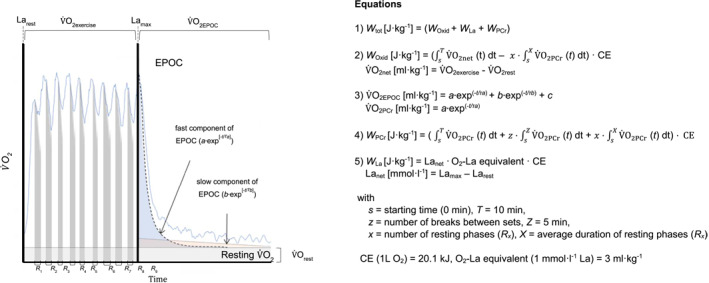
Illustration of the intermittent model approach of the PCr‐La‐O_2_ method, together with the corresponding equations to calculate the metabolic costs. The exponential function of the fast component at the end of the exercise was assessed by a bi‐exponential regression analysis of EPOC and applied to each resting phase (*R*_*x*_) during exercise.

Metabolic work was determined via indirect calorimetry from exercising O_2_ above rest, fast component of EPOC, and net La. Taking into consideration the fractional replenishment of high‐energetic muscular phosphates during resting phases between rallies or intervals, an intermittent approach accounted for the number and duration of breaks. The model and corresponding equations are provided in Figure 2. The total amount of metabolic costs (*W*_tot_) was calculated as the sum of oxidative (*W*_Oxid_), phosphagen (*W*_PCr_), and glycolytic (*W*_La_) metabolic work (Equation 1). *W*_Oxid_ was estimated from exercising $\dot{V}$ O_2_ above rest ($\dot{V}$ O_2net_) and a caloric equivalent for oxygen (CE) (Franchini, 2023; Stegemann, 1991) after subtracting the sum of $\dot{V}$ O_2_ that was attributed to PCr‐replenishment during the resting phases (Equation 2). Resting $\dot{V}$ O_2_ ($\dot{V}$ O_2rest_) was set according to a generally accepted value for a standing position (4.5 mL·kg^−1^· min^−1^) (Beneke et al., 2004) and CE of 20.1 kJ per 1 L of oxygen was chosen (Franchini, 2023; Stegemann, 1991). *W*_PCr_ was estimated from the fast component ($\dot{V}$ O_2PCr_) of EPOC ($\dot{V}$ O_2EPOC_), which was mathematically fitted using bi‐exponential non‐linear regression (Equation 3). As it is not possible to use the exponential modulation during the short breaks, for the intermittent model, PCr‐kinetics (*a*, *τ*_*a*_) from the end of drill/match EPOC curve (determined by Equation 3) were applied to the respective number and average duration of the breaks. Therefore, the number and duration of the resting phases were counted via video analyses, the integral of the modulated $\dot{V}$ O_2PCr_ was built for the average duration of the resting phases and the results were multiplied by the absolute number of rests for each data set (Equation 4). *W*_La_ was determined from the highest change in La (La_net_), an O_2_‐La‐equivalent, and CE (Equation 5). In multifeeding, La_net_ was determined from the initial increase during the first set (*T*_10_: 400 s, *T*_30_: 600 s, *T*_50_: 400 s) to consider continuous La breakdown during exercise (see Appendix). Assuming a distribution space of La close to 45% of body mass, an O_2_‐La‐equivalent of 3.0 mL·kg^−1^ mmol^−1^ was chosen (Di Prampero, 1981). Relative energy expenditure (*E*_tot_, *E*_Oxid_, *E*_PCr_, *E*_La_ in J·kg^−1^·min^−1^) was calculated by dividing the absolute metabolic costs (*W*_tot_, *W*_Oxid_, *W*_PCr_, *W*_La_) by bodyweight and the duration of the respective measurement period. The percentage contribution of each energy system was estimated by dividing the absolute amount of energy from a given system by the total amount of energy expenditure. The reliability of this approach was stated to be acceptable in applied settings (Kaufmann, Latzel, et al., 2022).

### 2.5 Statistical analyses

Excel software (Microsoft‐Office‐365) was used to calculate energy demands. SPSS statistical software (IBM Corp. Released 2015. IBM SPSS Statistics for Windows, Version 23.0. Armonk, NY: IBM Corp.) was used for EPOC fitting, excluding data with *r* ^2^ < 0.9 after data smoothing with a moving average of 10 s. The area under the curve was calculated with an online integration calculator using the trapezoidal rule. Jamovi (The Jamovi Project) was used to perform statistical analyses. Normality was assessed using the Shapiro–Wilk test. In the case of normal distribution, repeated analyses of variance (rANOVA) were used for comparisons between consecutive sets of match play and training protocols. Paired *t*‐tests were used for post‐hoc testing. The significance level was set at 5% (*p* < 0.05) and adjusted for post‐hoc testing using Tukey correction (*p*_tukey_ < 0.05). For non‐parametric data, Friedman and Wilcoxon tests were used instead. Descriptive statistics are reported as mean ± standard deviation, 95% confidence intervals (CI), and mean differences (MD). Effect sizes are given as *η* ^2^ (Cohen, 2013).

## 3 RESULTS

Video analyses revealed an average rally‐to‐rest ratio of 1:2, an active playing time of 10.3 ± 0.9 min, and average rally and rest durations of 6.9 ± 0.9 and 12.4 ± 1.8 s, respectively, during the training matches. While active playing time was comparable between *T*_*M*_ and the multifeeding drills (10 min), the predetermined rally‐to‐rest ratios were higher in the multifeeding drills (1:1). The temporal structure of the *T*_10_ protocol came closest to the average rally durations in *T*_*M*_, whereas *T*_30_ and *T*_50_ reflected rather occasional situations (3.0 ± 2.7% were >18 s). While average $\dot{V}$ O_2_ (*p* = 0.403), HR (*p* = 0.816), and RPE (*p* = 0.078) were comparable across all multifeeding drills, the respiratory exchange ratio (RER) and La_net_ significantly increased in the case of longer interval durations (*T*_50_ > *T*_30_ > *T*_10_; RER: *F* = 11.8, *p* < 0.001, *η* ^2^ = 0.260; La_net_: *F* = 14.6, *p* < 0.001, *η* ^2^ = 0.283)_._ The temporal characteristics of *T*_*M*_ are shown in Table 2, and descriptive statistics of all external and internal loads are presented in Table 3.

**TABLE 2:** Temporal and notational characteristics of *T*_*M*_ according to video analyses.

	Number of sets	Number of rallies	Rally duration [s]	Resting duration [s]	Rest between sets [min]	Rally to rest ratio	Active playing time [min]	Total playing time [min]
Total	2	84.3 ± 13.8	6.9 ± 0.9	12.4 ± 1.8	5	1:2	10.3 ± 0.9	30
Male	2	85.5 ± 11.0	7.5 ± 0.5	12.7 ± 2.2	5	1:2	10.6 ± 1.0	30
Female	2	82.0 ± 24.0	5.8 ± 0.2	11.7 ± 0.1	5	1:2	9.6 ± 0.4	30

**TABLE 3:** Descriptives of external loads, internal loads, and metabolic profiles.

		*T*_*M*_		*T*_10_		*T*_30_		*T*_50_	
		Mean ± SD	95% CI	Mean ± SD	95% CI	Mean ± SD	95% CI	Mean ± SD	95% CI
External loads									
Strokes [stroke·s^−1^]	Mean	0.9 ± 0.1	[0.9; 1.0]	0.6 ± 0.1	[0.6; 0.6]	0.6 ± 0.1	[0.5; 0.6]	0.5 ± 0.1	[0.5; 06]
Internal loads									
$\dot{V}$ O_2_ [ml·min^−1^ kg^−1^]	Mean	35.9 ± 5.2	[32.1; 39.9]	45.4 ± 5.8	[42.2; 48.5]	46.9 ± 7.7	[42.7; 51.0]	44.5 ± 5.6	[41.4; 47.6]
RER	Mean	0.88 ± 0.05	[0.85; 0.93]	0.91 ± 0.05	[0.88; 0.93]	0.93 ± 0.02	[0.91; 0.94]	0.96 ± 0.03	[0.93; 0.98]
La [mmol·L^−1^]	Peak	2.2 ± 1.1	[1.4; 3.0]	3.7 ± 1.9	[2.7; 4.8]	6.1 ± 1.9	[4.6; 7.8]	7.7 ± 2.2	[6.5; 8.8]
HR [bpm]	Mean	152 ± 11	[144; 161]	162 ± 12	[156; 169]	165 ± 12	[157; 172]	166 ± 10	[160; 172]
RPE [arbitrary unit]	Peak	14.8 ± 2.4	[12.7; 16.9]	15.9 ± 2.0	[14.8; 17.0]	17.0 ± 1.9	[16.0; 18.0]	17.3 ± 2.1	[16.0; 18.5]
Metabolic profiles									
*W*_tot_ [kJ]	Mean	1396 ± 349	[1138; 1655]	453 ± 104	[397; 510]	640 ± 162	[552; 729]	505 ± 119	[441; 570]
*W*_Oxid_ [kJ]	Mean	801 ± 241	[622; 980]	214 ± 57	[183; 245]	360 ± 96	[308; 413]	288 ± 69	[251; 326]
*W*_PCr_ [kJ]	Mean	589 ± 175	[459; 719]	232 ± 66	[196; 268]	267 ± 86	[220; 313]	160 ± 59	[128; 192]
*W*_La_ [kJ]	Mean	6 ± 5	[2; 11]	7 ± 4	[5; 10]	13 ± 6	[10; 17]	57 ± 24	[44; 71]
*W*_Oxid_ [%]	Mean	56.9 ± 8.6	[50.5; 63.3]	47.3 ± 7.7	[43.2; 51.5]	56.5 ± 6.2	[53.1; 59.9]	57.3 ± 6.0	[54.0; 60.5]
*W*_PCr_ [%]	Mean	42.7 ± 8.7	[36.3; 49.1]	51.1 ± 8.3	[46.5; 55.6]	41.2 ± 6.9	[37.6; 45.1]	31.2 ± 7.7	[27.0; 35.4]
*W*_La_ [%]	Mean	0.4 ± 0.6	[0.1; 0.7]	1.6 ± 1.0	[1.1; 2.1]	2.1 ± 1.0	[1.6; 2.7]	11.6 ± 4.8	[9.0; 14.2]
*E*_tot_ [J·kg^−1^·min^−1^]	Mean	676 ± 98	[603; 749]	1020 ± 160	[932; 1107]	985 ± 173	[890; 1079]	1266 ± 194	[1160; 1371]
*E*_Oxid_ [J·kg^−1^·min^−1^]	Mean	388 ± 92	[320; 456]	485 ± 115	[423; 5547]	556 ± 118	[492; 620]	724 ± 127	[654; 793]
*E*_PCr_ [J·kg^−1^·min^−1^]	Mean	285 ± 55	[244; 326]	518 ± 100	[464; 572]	407 ± 97	[355; 460]	396 ± 117	[332; 460]
*E*_La_ [J·kg^−1^·min^−1^]	Mean	3 ± 3	[1; 5]	17 ± 10	[11; 22]	21 ± 10	[16; 26]	146 ± 61	[113; 179]

*Note*: Descriptives are displayed as mean ± standard deviation (SD), with lower and upper limit of the 95% confidence interval (CI) displayed as [lower; upper] for the training match (*T*_*M*_) and the multifeeding protocols (*T*_10_, *T*_30_, *T*_50_). Absolute values refer to a measurement period of 900s in *T*_*M*_, 600s in *T*_30_, and 400s in *T*_10_ and *T*_50_.

Abbreviations: *E*_La_, relative energy expenditure from anaerobic glycolysis; *E*_Oxid_, relative energy expenditure from oxidative metabolism; *E*_PCr_, relative energy expenditure from phosphagen metabolism; *E*_tot_, relative energy expenditure; HR, heat rate; La, blood lactate concentrations; RER, respiratory exchange ratio; RPE, rate of perceived exertion according to Borg scale (6–20); $\dot{V}$ O_*2*_, oxygen consumption; *W*_La_, energy from anaerobic glycolysis in kJ and percentual contribution of these energetic systems in %; *W*_Oxid_, energy from oxidative metabolism; *W*_PCr_, energy from phosphagen metabolism; *W*_tot_, total metabolic costs.

**FIGURE 3:**
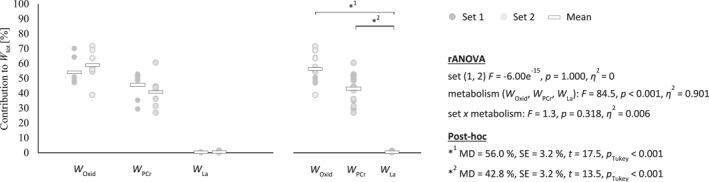
Metabolic profile of badminton match play and comparison between consecutive sets. Each dot represents the measurement of a single player. * Mark significant post‐hoc tests. Results of the repeated analyses of variance as well as significant post‐hoc tests are provided next to the graphs.

**FIGURE 4:**
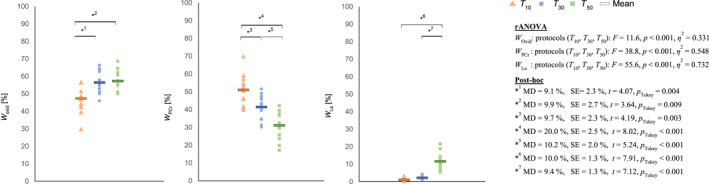
Metabolic profile of the badminton multifeeding drills and comparison between training protocols for *W*_Oxid_, *W*_PCr_ and *W*_La_, respectively. Each dot represents the measurement of a single player. * Mark significant post‐hoc tests. Results of the repeated analyses of variance as well as significant post‐hoc tests are provided next to the graphs.

In T_*M*_, the total metabolic costs were predominantly covered by the oxidative and phosphagen energetic systems, whereas anaerobic glycolysis was significantly less involved (*F* = 84.5, *p* < 0.001, *η* ^2^ = 0.901). As illustrated in Figure 3, the relative contributions of aerobic and anaerobic energy systems remained consistent between consecutive sets of match play (*p* = 0.318). In the multifeeding drills, *E*_tot_, *E*_Oxid_, and *E*_La_ were comparable for *T*_10_ and *T*_30_ but were significantly higher in *T*_50_ (*E*_tot_: *F* = 44.4, *p* < 0.001, *η* ^2^ = 0.352; *E*_Oxid_: *F* = 27.6, *p* < 0.001, *η* ^2^ = 0.429; *E*_La_: *F* = 58.3, *p* < 0.001, *η* ^2^ = 0.748), while *E*_PCr_ was comparable for *T*_30_ and *T*_50_ but significantly higher in *T*_10_ (*F* = 17.4, *p* < 0.001, *η* ^2^ = 0.228). The percentage share of *W*_Oxid_ was significantly lower in *T*_10_ but comparable in *T*_30_ and *T*_50_ (*F* = 11.6, *p* < 0.001, *η* ^2^ = 0.331). The percentage share of *W*_PCr_ was highest in *T*_10_, followed by *T*_30_ and lowest in *T*_50_ (*F* = 38.8, *p* < 0.001, *η* ^2^ = 0.548) and the relative share of *W*_La_ was significantly higher in *T*_50_, while no differences were observed between *T*_10_ and *T*_30_ (*F* = 55.6, *p* < 0.001, *η* ^2^ = 0.732). Descriptive statistics of absolute metabolic costs, energy expenditure related to body mass and time, and the percentage contribution of the given energy systems are shown in Table 3 (for the whole sample) and Table 4 (for male and female players, separately). Figure 4 presents the percentual contribution of each energy system in the different multifeeding drills.

**TABLE 4:** Descriptives of external loads, internal loads, and metabolic profiles for male and female players separately.

		*T*_*M*_		*T*_10_		*T*_30_		*T*_50_	
		Mean ± SD	95% CI	Mean ± SD	95% CI	Mean ± SD	95% CI	Mean ± SD	95% CI
*W*_aer_ [%]	Male	55.3 ± 8.0	[48.2; 62.3]	45.4 ± 7.6	[40.4; 50.3]	55.3 ± 6.2	[51.3; 59.3]	56.5 ± 5.4	[53.0; 60.1]
	Female	61.0 ± 12.0	[44.3; 77.7]	51.8 ± 6.7	[45.1; 58.4]	59.1 ± 6.5	[52.8; 65.5]	58.9 ± 7.7	[51.4; 66.5]
*W*_PCr_ [%]	Male	44.2 ± 8.1	[37.1; 51.4]	53.2 ± 8.1	[47.9; 58.5]	42.6 ± 6.7	[38.2; 47.1]	33.8 ± 5.0	[30.6; 37.1]
	Female	38.8 ± 12.0	[22.1; 55.5]	46.4 ± 7.8	[38.8; 54.0]	38.6 ± 7.4	[31.4; 45.8]	25.1 ± 10.1	[15.2; 35.0]
*W*_La_ [%]	Male	0.5 ± 0.3	[0.2; 0.8]	1.5 ± 0.9	[0.9; 2.0]	2.1 ± 1.0	[1.4; 2.7]	9.6 ± 3.1	[7.6; 11.7]
	Female	0.1 ± 0.0	[0.1; 0.1]	1.9 ± 1.2	[0.7; 3.1]	2.3 ± 0.9	[1.4; 3.2]	16.0 ± 5.4	[10.7; 21.2]
*E*_tot_ [J·kg^−1^·min^−1^]	Male	670 ± 118	[566; 773]	1094 ± 124	[1014; 1175]	1064 ± 144	[970; 1175]	1342 ± 173	[441; 570]
	Female	690 ± 42	[633; 749]	851 ± 90	[764; 940]	804 ± 53	[753; 940]	1094 ± 121	[441; 570]
E_Oxid_ [J·kg^−1^·min^−1^]	Male	374 ± 94	[291; 456]	503 ± 124	[422; 584]	592 ± 122	[512; 672]	758 ± 122	[678; 837]
	Female	424 ± 108	[273; 575]	444 ± 93	[353; 536]	476 ± 53	[424; 527]	646 ± 118	[531; 762]
*E*_PCr_ [J·kg^−1^·min^−1^]	Male	292 ± 57	[243; 342]	575 ± 52	[541; 609]	450 ± 76	[401; 500]	453 ± 80	[401; 506]
	Female	265 ± 67	[173; 358]	391 ± 38	[354; 427]	311 ± 66	[246; 376]	268 ± 82	[188; 349]
*E*_La_ [J·kg^−1^·min^−1^]	Male	4 ± 3	[1; 6]	17 ± 11	[10; 23]	22 ± 11	[15; 29]	131 ± 52	[97; 165]
	Female	1 ± 0	[1; 1]	17 ± 12	[5; 28]	28 ± 7	[11; 25]	179 ± 75	[106; 252]

*Note*: Descriptives are displayed as mean ± standard deviation (SD), with lower and upper limit of the 95% confidence interval (CI) displayed as [lower; upper] for the training match (*T*_*M*_) and the multifeeding protocols (*T*_10_, *T*_30_, *T*_50_).

Abbreviations: *E*_La_, relative energy expenditure from anaerobic glycolysis; *E*_Oxid_, relative energy expenditure from oxidative metabolism; *E*_PCr_, relative energy expenditure from phosphagen metabolism; *E*_tot_, relative energy expenditure; *W*_La_, percentual contribution of anaerobic glycolysis; *W*_Oxid_, percentual contribution of oxidative metabolism; *W*_PCr_, percentual contribution of phosphagen metabolism.

**FIGURE 5:**
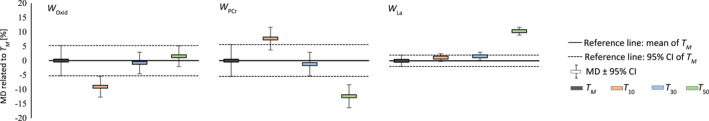
Mean difference (MD) of the relative share of oxidative (*W*_Oxid_), phosphagen energetic (*W*_PCr_) and anaerobic glycolytic (*W*_La_) metabolic costs of the multifeeding drills (*T*_10,_ *T*_30_, *T*_50_) in relation to match play (*T*). Average of *T*_*M*_ is set as MD = 0% and upper and lower limits of 95% CI of *T*_*M*_ are displayed as reference lines (dotted lines), for *W*_Oxid_, *W*_PCr_, and *W*_La_, respectively.

**FIGURE 6:**
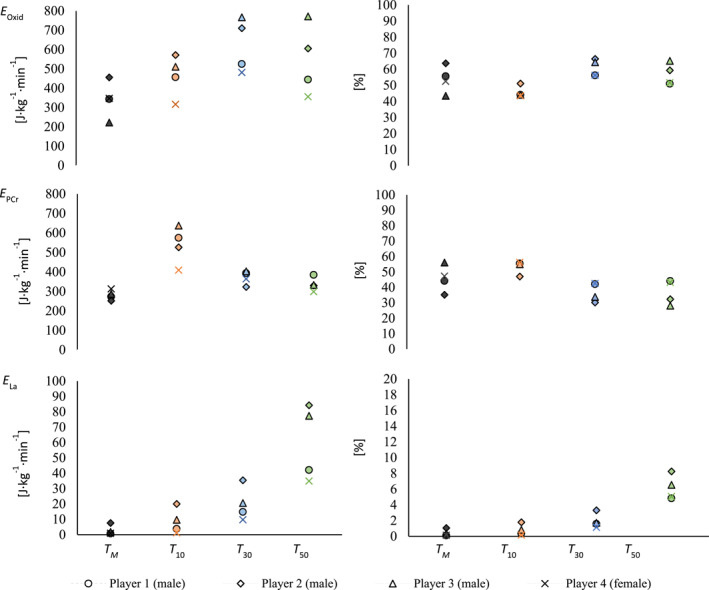
Individual comparison of relative energy expenditure and percentual contribution of the different metabolic systems between *T*, *T*_10_, *T*_30_ and *T*_50_ for the players that absolved all testing sessions (*T*_*M*_ and the three multifeeding drills).

Due to the small sample size of players who participated in both the *T*_*M*_ and the multifeeding drills, comparisons between match play and training drills are based on descriptive analysis only. Accordingly, the metabolic profile of *T*_30_ came closest to that of T_*M*_ (MD_(*W*Oxid)_ = 1.0%, MD_(*W*PCr)_ = −2.7%, MD_(*W*La)_ = 1.6%), while *T*_10_ required lower *W*_Oxid_ (MD = −8.2%) but higher *W*_PCr_ (MD = 6.9%), and *T*_50_ led to lower *W*_PCr_ (MD = −13.0%) but higher *W*_La_ (MD = 11.1%). The relative contribution of the energy systems during multifeeding related to *T*_*M*_ are illustrated in Figure 5. Figure 6 illustrates the individual comparison of the energetics between *T*_*M*_, *T*_10_, *T*_30_, and *T*_50_ for the players who participated in all sessions. Individual metabolic profiles for all participants and each testing session are provided in detail in the Appendix.

## 4 DISCUSSION

The primary objective of this study was to ascertain and compare the metabolic profiles of elite players during badminton match play and typical badminton on‐court drills. The results revealed that energy in badminton is predominantly derived from oxidative and phosphagen energetic pathways, with anaerobic glycolytic metabolism contributing only minimally. Although this applies in principle to matches as well as to multifeeding drills, the metabolic costs of drills differ significantly depending on the interval duration. Concretely, shorter intervals (10 s) could be associated with a higher percentage contribution of the phosphagen metabolism, while longer intervals (50 s) induced greater lactic stress. The metabolic profile of mid‐length intervals (30 s) came closest to that of match play.

Badminton matches require significant and dynamically changing energy provision due to multiple high‐intensity activities, including rapid accelerations, abrupt stops, and quick changes in direction, jumps, and striking series. Previous research has underscored the intensity of these activities, pointing to a substantial reliance on both oxidative and phosphagen energy systems (Faude et al., 2007; Phomsoupha et al., 2015). The current study affirms and extends these observations, revealing the noteworthy contribution of oxidative (56.9 ± 8.6%) and phosphagen (42.7 ± 8.7%) energy systems, with only minimal engagement of anaerobic glycolysis (0.4 ± 0.6%) in elite players' match play. These findings confirm the evidence that, in badminton, metabolic costs during short rallies are predominantly sourced from high energetic phosphates, while resting phases are primarily fueled by the oxidative metabolism (Beneke et al., 2004). Considering that the limited stores of adenosintriphosphat (ATP) and PCr are depleted after 6–8 s (Dunst, 2020), higher oxidative capacity is necessary to replenish these reserves during breaks, ensuring a sustained high energetic flux throughout the entire match (Beneke et al., 2004; Edel et al., 2019; Hargreaves et al., 2020; Phomsoupha et al., 2015). Also, a higher reliance on anaerobic glycolysis during longer rallies or after short breaks seems reasonable, as PCr stores are probably fully depleted then. La concentrations, which tend to be rather low, therefore appear to result from an equilibrium between La production and clearance rate rather than from the absence of anaerobic glycolysis (Hargreaves et al., 2020).

In the assessment of multifeeding drills, all protocols essentially reflect the metabolic demands of match play but exhibit significant variations depending on rally and rest durations. Shorter intervals (10 s) exhibited a greater contribution from the phosphagen energetic pathway, while glycolysis significantly gained impact with increasing interval durations (50 s). Even though, from a temporal perspective 10 s intervals are most closely aligned with the average rally duration in matches (6.9 ± 0.9 s), 30 s intervals more accurately reflect the corresponding metabolic demands. This observation is likely attributed to the higher rally‐to‐rest ratio during matches (2:1 vs. 1:1 in multifeeding), which allows for better recovery between the subsequent bouts but also leads to longer periods fueled by oxidative energy systems. Considering that, in this context, the current study emphasizes the significance of rapid PCr replenishment as a key metabolic predictor of badminton performance and noting that maximum capacity of aerobic power ($\dot{V}$ O_2max_) was identified as a principal predictor for rapid PCr replenishment (McMahon et al., 2002), training regimes focusing on improving $\dot{V}$ O_2max_ might be highly beneficial for badminton players. Higher aerobic capacity might further lead to faster oxygen on‐set kinetics (Jones et al., 2000), less reliance on anaerobic glycolysis, and less H^+^ accumulation, which in turn would lead to more rapid PCr replenishment (Tomlin et al., 2001). In conclusion, players may benefit from enhancing repeated sprint ability by choosing short but very intense intervals. At the same time, the use of 30 s intervals may be reasonable when training loads closely align with competition‐specific metabolic demands, particularly during specific preparation phases for major competitions throughout the year. Although anaerobic glycolysis was shown not to be a primary source of energy during badminton match play in quantitative terms, anaerobic lactic capacity may still become relevant in certain, probably match‐deciding, situations. Thus, players with poor La tolerance or low La clearance rates could also gain advantages from selecting longer intervals.

Noting that previous research has found sex‐related differences in energy system contribution (Fu et al., 2021; Kaufmann, Ziegler, et al., 2022; Tortu et al., 2024), and acknowledging sex‐related differences in accelerations, rally and rest durations, and the number of strokes in badminton match play (Fernandez‐Fernandez et al., 2013; Rojas‐Valverde et al., 2020), disparities in the metabolic demands on male and female badminton players are conceivable. In the current study the male players tended to exhibit higher amounts of total energy expenditure and energy derived from muscular phosphates, while the female players tended toward a higher percentage contribution of oxidative energy systems and anaerobic glycolysis. These observations could be attributed to male players' overall higher aerobic capacity, higher anaerobic threshold, and higher PCr storage capacity, influenced by their greater lung volume, cardiac output, and levels of hemoglobin and muscle mass, as well as by their distinctive fiber type distribution (higher proportion of type I fibers in women and more type IIA and IIX fibers in males) (Faude et al., 2007; Hargreaves et al., 2020; Lundsgaard et al., 2014; Tai et al., 2022; Tortu et al., 2024). The greater reliance on oxidative and glycolytic systems in female players could be a compensatory mechanism for the discrepancy in muscular PCr storage capacity. These assumptions basically correspond with the findings of previous investigations (Fu et al., 2021; Kaufmann, Ziegler, et al., 2022; Tortu et al., 2024), but, based on the results of the current study, are speculative and require further verification by statistical analyses. Moreover, in some sports competition requirements and rules may differ for males and females, and thus it is difficult to draw unambiguous conclusions on sex‐related differences (Kaufmann, Ziegler, et al., 2022). Therefore, to draw valid and reliable conclusions, further evidence is needed that is (1) derived from a larger sample size, (2) addresses other individual influencing factors, such as body composition, fitness level, and playing style, and (3) accounts for external loads that might also impair energetic demands (e.g., strokes, jumps, accelerations, etc.).

To the best of our knowledge, this is the first study that has applied an intermittent model of the PCr‐La‐O_2_ method to determine the metabolic profile of elite badminton players. In contrast to a recent study that, utilizing a continuous model, reported the predominant contribution of aerobic metabolism (94.2%) in badminton match play (Fu et al., 2021), the results of the present study emphasize the substantial role of muscular PCr as an equally crucial energy source, revealing the limitations of continuous models in studies of intermittent exercises (Beneke et al., 2004; Dunst, 2020; Latzel et al., 2018). In line with this, previous research focusing on basketball (Latzel et al., 2018) and squash (Dunst, 2020) has already demonstrated that adopting a continuous model to study these sports led to an underestimation of the contribution of the phosphagen system, arising from the failure to consider PCr replenishment during breaks. Model assumption checks with the present data corroborated these insights, revealing significant disparities in oxidative and phosphagen provided energy shares when using a continuous calculation model instead (see Appendix A). In conclusion, the previous study's emphasis on oxidative energy systems can be attributed to the inaccurate association of the O_2_ requirement for PCr replenishment with the energy directly provided during matches. These findings underscore the importance of adopting an intermittent model to accurately assess energy system contributions in intermittent sports. Nevertheless, oxidative capacity remains pivotal in badminton, particularly concerning PCr replenishment.

### 4.1 Limitations

Considering the inherent fluctuations in the underlying energetics, the modeling approach still has some limitations. Notably, the average loads of match play may not reflect occasional intensity peaks, and the breakdown of La cannot be adequately assessed through single measurements. Moreover, a heightened adrenergic response may elevate glycolytic flux during official competitions, resulting in higher La compared to training matches (Faude et al., 2007; Rampichini et al., 2018; Zagatto et al., 2018). Consequently, it is crucial to acknowledge the potential underestimation of the anaerobic glycolytic energy supply, particularly during longer measurement periods (see Appendix). Furthermore, it is reasonable to assume that the fitness status of different individuals and of the same individual at different phases of the season will vary. This influences their metabolic capacities and, therefore, individual responses to different exercise protocols. Thus, adaptations to training programs should always be evaluated on an individual level using appropriate performance testing. However, the major limitation of this study might be the limited sample size. As the study aimed to be representative for elite level of performance, only players at the highest national level (Olympic, perspective and junior squad) could be included; therefore, the results are applicable to this specific subgroup of badminton players.

### 4.2 Practical applications

This study underscores the necessity of thoughtful selection of drill prescriptions in badminton, emphasizing the need to tailor training according to individualized goals and to structure long‐term training specifically to achieve these goals. Thus, the metabolic demands of match play serve as the only conceivable orientation for appropriate exercise selection. Accordingly, the present analyses highlight the importance for elite badminton players of possessing a well‐developed aerobic and anaerobic alactic endurance capacity to sustain high energetic flux and, consequently, high performance during competition. A comprehensive approach to training, encompassing highly specific on‐court conditioning drills, such as multifeeding, but also including less specific or less complex conditioning drills (e.g., repeated sprinting exercises), should be considered to develop these capacities effectively. Regarding multifeeding drills, the present study suggests the potential superiority of the 10 s protocol in enhancing the badminton‐specific requirement for rapid PCr replenishment, as this protocol showed the highest contribution of the phosphagen metabolic system. Conversely, training protocols featuring a longer interval duration (e.g., 50 s) could also enhance PCr replenishment as they are effective for improving La tolerance and clearance rates and probably the most efficient for enhancing $\dot{V}$ O_2max_. However, as sport‐specific on‐court drills are limited by coordinative aspects, they usually do not allow for supramaximal intensities. Therefore, less specific or less complex conditioning programs could serve as useful additions to meet the specific energetic requirements of badminton training. Repeated sprinting exercises, for instance, have been proven to be effective in increasing performance in badminton players due to enhanced aerobic capacity and accelerated recovery ability (Liu et al., 2021). Furthermore, future research could also address oral supplementation with creatine, as it promises enhancements in resting PCr capacity, PCr resynthesis rate, and La buffering (Ferrauti et al., 2003; Hargreaves et al., 2020). With regard to long‐term training periodization, training methods should become progressively more specific to the requirements of major competitions. This involves starting from nonspecific repetitive sprinting exercises to achieve the greatest adaptations of $\dot{V}$ O_2max_, advancing to on‐court drills to improve specific capabilities (e.g., 10 s intervals to improve the phosphagen energetic system and 50 s protocols to enhance lactic capacity), and peaking with activities that are the most proximate to those used in actual game, such as 30 s intervals in multifeeding drills. Future interventional studies are necessary to verify these suggested reasonable adaptations to long‐term, energy system–specific, on‐court training.

## 5 CONCLUSION

The present study underscores the pivotal role of phosphagen metabolism in badminton, highlighting the critical need for rapid PCr replenishment to prevent performance decline attributed to increasing acidosis and the resultant inhibition of further glycolysis. While oxidative capacity remains vital for PCr replenishment, occasional reliance on anaerobic glycolysis may be important in certain situations. Multifeeding drills essentially mirror the metabolic demands of matches but exhibit variations depending on the duration of rallies and rest periods. As the interval length increases, the dominance of oxidative and anaerobic glycolytic metabolism rises, while the proportion of energy from PCr storage decreases. Thus, 10 s intervals may be the most suitable to address phosphagen energetic systems, while 50 s intervals target lactic capacities, and 30 s intervals most closely reflect the metabolic demands of match play. Overall, the present study draws attention to the importance of a well‐developed aerobic and phosphagen energetic endurance capacity in elite‐level badminton and shows that different interval (and rest) durations address different metabolic pathways in on‐court training. Accordingly, training content and, especially protocol prescriptions should be chosen wisely to address individual training goals and align with a long‐term training periodization perspective.

## AUTHOR CONTRIBUTION

Antonia Edel, Thimo Wiewelhove, and Alexander Ferrauti have made substantial contributions to conception and design. Antonia Edel was responsible for acquisition of data, data analysis and interpretation of data. Jo‐Lâm Vuong, Thimo Wiewelhove, and Alexander Ferrauti were involved in interpretation of the data. Sebastian Kaufmann and Olaf Hoos were consulted for methodical approach and data analysis. All authors have been involved in drafting the manuscript or revising it critically. All authors participated sufficiently in the work, have given final approval of the version to be published, take public responsibility for appropriate portions of the content, and agreed to be accountable for all aspects of the work in ensuring that questions related to the accuracy or integrity of any part of the work are appropriately investigated and resolved.

## CONFLICT OF INTEREST STATEMENT

The authors have stated explicitly that there are no conflicts of interest in connection with this article.

## PATIENT CONSENT STATEMENT

All participants were informed about the testing procedures, data policy, and potential risks of the study and gave their written informed consent to voluntarily participate.

## PERMISSION TO REPRODUCE MATERIAL FROM OTHER SOURCES

The article contains no material that has been reproduced from any other sources.

## CLINICAL TRIAL REGISTRATION

Not applicable.

## Data Availability

The data that support the findings of this study are available from the corresponding author upon reasonable request.

## APPENDIX A

### A.1 Model assumption checks

Different calculation models of the PCr‐La‐O_2_ method have been discussed in the literature. So far, it is well established that for high‐intensity exercises, bi‐exponential regression analysis is superior to linear or mono‐exponential EPOC fitting. Recent research suggested a model for intermittent exercises that, unlike the conventional approach (continuous model), accounts for replenishment of PCr during resting periods (intermittent model) (Latzel et al., 2018). In addition, in high‐intensity intermittent exercises that rely on the lactic metabolic pathway, the highest rise in blood La concentrations typically occurs at the beginning of the exercise. After that, blood La concentrations usually reach a plateau, or at least there is a reduction in their gradient. Physiologically, this is reasonable due to the bi‐carbonate buffering system and continuous La metabolization throughout an exercise and should not be interpreted as a termination of the anaerobic glycolysis. As the area under the O_2_ curve continuously rises, this might lead to an underestimation of the anaerobic lactic metabolic pathways, which increases with ongoing measurement duration. For this reason, the analyzed timeframe may be decisive. For this investigation only the initial phase of the multifeeding drills (*T*_10_: 400 s, *T*_30_: 600 s, and *T*_50_: 400 s) was considered for the calculation of the metabolic profiles (complete vs. initial phase model). However, to compare the results to previous findings and to obtain more insight into possible sources of systematic errors in the underlying calculation models, both the continuous and intermittent model results (Table A1) as well as intermittent model results of the complete and the initial phase model (Table A2) are provided in this appendix.

**TABLE A1:** Comparison between continuous and intermittent calculation model.

	Continuous	Intermittent	rANOVA			
	Mean ± SD	Mean ± SD	MD	SE	*p*	*η* ^2^
*W*_Oxid_ [%]	96.9 ± 0.91	56.9 ± 8.6	42.9	3.4	<0.001	0.927
*W*_PCr_ [%]	2.7 ± 0.7	42.7 ± 8.7	42.9	3.4	<0.001	0.927
*W*_La_ [%]	0.4 ± 0.6	0.4 ± 0.6	0	–	–	–

*Note*: Descriptives are displayed as mean ± standard deviation (SD), Inference statistical results of the repeated measures analyses of variance (rANOVA) are displayed as mean difference (MD), standard error (SE), *p*‐values, and effect sizes (*η* ^2^).

**TABLE A2:** Comparison of metabolic profiles calculated for complete drill duration (1200 s for *T*_10_, *T*_30_ and *T*_50_) or a predefined initial phase of the drill (*T*_10_: 400 s, *T*_30_: 600 s, *T*_50_: 400 s).

	Complete	Initial phase	rANOVA			
	Mean ± SD	Mean ± SD	MD	SE	*p*	*η* ^2^
*T*_10_						
*W*_Oxid_ [%]	48.9 ± 7.1	47.3 ± 7.7	−1.5	0.7	0.043	0.012
*W*_PCr_ [%]	50.3 ± 7.5	51.1 ± 8.3	0.8	0.7	0.304	0.003
*W*_La_ [%]	0.8 ± 0.6	1.6 ± 1.0	0.8	0.2	<0.001	0.204
*T*_30_						
*W*_Oxid_ [%]	58.1 ± 6.0	56.5 ± 6.3	−1.7	0.2	<0.001	0.020
*W*_PCr_ [%]	40.2 ± 6.3	41.4 ± 6.9	1.2	0.3	0.002	0.009
*W*_La_ [%]	1.7 ± 0.7	2.1 ± 1.0	0.4	0.2	0.015	0.068
*T*_50_						
*W*_Oxid_ [%]	59.9 ± 8.2	57.3 ± 6.0	−2.6	1.3	0.073	0.035
*W*_PCr_ [%]	33.5 ± 8.7	31.2 ± 7.7	−2.4	1.0	0.031	0.022
*W*_La_ [%]	6.6 ± 2.5	11.6 ± 4.8	5.0	0.8	<0.001	0.317

*Note*: Descriptives are displayed as mean ± standard deviation (SD), Inference statistical results of the repeated measures analyses of variance (rANOVA) are displayed as mean difference (MD), standard error (SE), *p*‐values, and effect sizes (*η* ^2^).
